# Mindfulness-based interventions for gambling disorder: A systematic review

**DOI:** 10.1556/2006.2025.00100

**Published:** 2026-02-03

**Authors:** Atanas Tannous, Zsolt Demetrovics, Bhavya Chhabra, Alexander Logemann, Andrea Czakó, Mark D. Griffiths, Attila Szabo

**Affiliations:** 1 Doctoral School of Psychology, ELTE Eötvös Loránd University, Budapest, Hungary; 2 Institute of Psychology, ELTE Eötvös Loránd University, Budapest, Hungary; 3 Flinders University Institute for Mental Health and Wellbeing, College of Education, Psychology and Social Work, Flinders University, Bedford Park, SA, Australia; 4 Centre of Excellence in Responsible Gaming, University of Gibraltar, Gibraltar; 5 Institute of Health Promotion and Sport Sciences, Faculty of Education and Psychology, ELTE Eötvös Loránd University, Budapest, Hungary; 6 Doctoral School of Education, Faculty of Education and Psychology, ELTE Eötvös Loránd University, Budapest, Hungary; 7 International Gaming Research Unit, Psychology Department, Nottingham Trent University, 50 Shakespeare Street, Nottingham, UK; 8 Faculty of Health and Sport Sciences, Széchenyi István University, Győr, Hungary; 9 Department of Clinical, Neuro and Developmental Psychology, Vrije Universiteit Amsterdam, Amsterdam, Netherlands

**Keywords:** mindfulness, mindfulness-based interventions (MBI), treatment, gambling disorder, problem gambling

## Abstract

**Background and Aims:**

Gambling disorder (GD) presents significant psychological, financial, and social consequences. Mindfulness-based interventions (MBIs) have emerged as promising adjunctive treatments. However, the evidence base remains heterogeneous. The present systematic review evaluated the current empirical literature on MBIs for GD, focusing on their efficacy, methodological quality, and limitations.

**Methods:**

*Scopus, PubMed, Web of Science, EBSCO,* and *PsycINFO* were searched for English-language studies published between 2012 and April 2025. The final sample comprised 12 studies: five randomized controlled trials, one controlled pilot study, two repeated-measures studies, one mixed-methods study, and three single-group pretest–posttest studies. A narrative synthesis evaluated MBI impacts.

**Results:**

MBIs consistently reduced gambling frequency and cravings while enhancing psychological outcomes. Studies combining mindfulness with cognitive behavioral therapy showed significant declines in problem gambling behavior. Psychological distress and cravings also decreased notably across interventions. The mindfulness components employed varied in focus and application, adding nuance to outcome interpretation. However, it remains unclear to what extent the observed effects can be attributed to mindfulness-specific mechanisms.

**Discussion and Conclusions:**

MBIs show promise as a complementary treatment for gambling disorders, although small sample sizes and methodological limitations suggest a need for more robust research.

## Introduction

Gambling, defined as wagering something of financial value on an outcome with uncertain results (Griffiths, 1995), is increasingly accessible via digital and land-based platforms (Akçayır, Nicoll, & Baxter, 2023; Chóliz, 2016). While many engage recreationally, global prevalence estimates suggest that 0.12–5.8% of the population meet criteria for gambling disorder, with 0.23% of adults seeking treatment (Calado & Griffiths, 2016; Gabellini, Lucchini, & Gattoni, 2023). A 2024 global meta-analysis reported that 8.7% of adults engage in at-risk gambling and 1.4% meet criteria for problem gambling (Tran et al., 2024). Rates vary globally, with low- and middle-income countries—experiencing growing harms due to the spread of online gambling (Ssewanyana & Bitanihirwe, 2018; World Health Organization, 2024). For example, in sub-Saharan Africa, youth engagement is high, with Western African students reporting 57% lifetime and 78% current gambling, and Southern African community samples reporting 68% lifetime and 41% regular gambling (Bitanihirwe & Ssewanyana, 2021). Participation is also high in East/Southeast Asia (approximately half of all adults gamble; ∼2% problematically) and comparatively lower in South America (30–35% participation; 2–4% problematically; Tran et al., 2024). Many affected individuals do not seek help due to barriers (Lischer, Schwarz, Wallimann, Jeannot, & Mathys, 2023), and studies on alternative treatment options remain scarce (Rolvien, Buddeberg, Gehlenborg, Borsutzky, & Moritz, 2024).

In the fifth edition of the *Diagnostic and Statistical Manual of Mental Disorders* (DSM-5), gambling disorder (GD) was reclassified from an impulse control disorder to a behavioral addiction, characterized by recurrent gambling behavior that causes functional impairment and distress (American Psychiatric Association, 2013; Hodgins, Stea, & Grant, 2011). Severity is based on endorsement of 4–9 diagnostic criteria, while those meeting 2–3 criteria are considered “problem gamblers” (Loo, Kraus, & Potenza, 2019). As a sub-clinical classification, problem gambler refers to individuals who endorse two or three of the nine diagnostic criteria (Loo et al., 2019).

The Pathways Model of problem gambling (Blaszczynski & Nower, 2002; Nower, Blaszczynski, & Anthony, 2022) delineates three subtypes of gamblers—behaviorally conditioned, emotionally vulnerable, and antisocial-impulsive—each with distinct mechanisms. The model emphasizes that all problem gambling begins with gambling availability but diverges according to different underlying etiologies, supporting tailored clinical interventions (Nower et al., 2022). Subsequent research has refined these categories to better target intervention strategies, with emotional dysregulation, cognitive distortions, and impulsivity identified as key drivers of disordered gambling (Bonnaire & Billieux, 2022; Canale, Vieno, Griffiths, Rubaltelli, & Santinello, 2015; Nower et al., 2022).

Disordered gambling prevalence rates are higher among males (Potenza et al., 2019), minority groups (Potenza et al., 2019; Welte, Wieczorek, Barnes, & Tidwell, 2006), those experiencing intimate partner violence, those with a history of gambling in the family, and those with lower education levels (Potenza et al., 2019). Several factors, including socioeconomic status (van der Maas, 2016), gender (Calado & Griffiths, 2016; Hing, Russell, Tolchard, & Nower, 2016), cultural background (Subramaniam et al., 2015), mental health (van der Maas, 2016), and social support (Penfold & Ogden, 2024) influence the way in which GD presents itself. Additionally, gambling disorder is frequently accompanied by high rates of comorbid psychiatric conditions, including mood disorders such as major depression (Sharma & Weinstein, 2025), substance use disorders including alcohol and drug use (Lorains, Cowlishaw, & Thomas, 2011), anxiety disorders, and personality disorders, which complicate diagnosis and treatment (Sharma & Weinstein, 2025). Given the wide variability in how individuals experience, express, and suffer from gambling-related problems, a single, universally applicable solution to the issue may not be suitable for all gamblers who seek treatment (Petry, 2005).

Cognitive-behavioral therapies (CBTs) remain the most widely used treatments for gambling disorder (Potenza et al., 2019). Their cognitive elements target the thoughts, attitudes, and beliefs that drive gambling, including challenging distortions such as overconfidence in detecting “winning systems,” ritualistic behaviors presumed to influence outcomes, and other statistically inaccurate beliefs (Cowlishaw et al., 2012). The behavioral elements focus on identifying external triggers, rehearsing alternative responses to high-risk cues, and building rewarding, non-gambling activities into daily life (Potenza et al., 2019). Within this family, cognitive therapy (CT) explicitly corrects irrational cognitions, while exposure therapy (ET) with response prevention deliberately elicits urges via gambling cues or simulated environments and then coaches patients to resist acting on them, progressively strengthening self-control (Bergeron, Giroux, Chrétien, & Bouchard, 2022). While considered one of the most efficient interventions, CBT is frequently associated with non-response and high relapse rates (Chrétien, Giroux, Goulet, Jacques, & Bouchard, 2017). Additionally, CBT has not been found to significantly reduce emotional distress, a core feature of disordered gambling (Kang, Fairbairn, & Ariss, 2019). Moreover, the effect of CBTs on these outcomes could be overestimated, and CBTs might not be reliably efficacious for everyone seeking treatment for problem gambling and gambling disorder (Pfund et al., 2023). These limitations have led to growing interest in alternative approaches that target emotional regulation and attentional processes more directly. One such emerging approach is mindfulness.

Mindfulness is defined as “the awareness that emerges through paying attention on purpose, in the present moment, and non-judgmentally to the unfolding of experience moment by moment” (Kabat-Zinn, 2003, p. 145). Its origins trace back to the Pali word ‘sati’ (or ‘smriti’ in Sanskrit). Sati is the cultivation of a receptive state of consciousness and a present-moment awareness and is a component of various Buddhist traditions (Levman, 2018). Scientifically, mindfulness has been conceptualized as a trainable cognitive ability, a spiritual practice, and a mental quality linked to well-being (Davidson & Kaszniak, 2015; Grossman & Van Dam, 2011; Van Dam et al., 2018).

Two major frameworks have guided the understanding of mindfulness mechanisms: Monitor and Acceptance Theory (MAT) and the Intention-Attention-Attitude (IAA) model. MAT proposes that mindfulness involves attentional monitoring and acceptance, with the latter reduces emotional reactivity and improves regulation (Lindsay & Creswell, 2017). The IAA model describes mindfulness as a recursive process involving intentionality, attention, and attitude. This leads to a shift known as ‘reperceiving’, which enhances flexibility in thought and emotion (Shapiro, Carlson, Astin, & Freedman, 2006). Across models, mindfulness fosters openness and non-judgmental awareness—capacities central to self-regulation (Bishop et al., 2004).

In recent years, this practice has become increasingly popular within healthcare, education, and workplace environments and among varied demographics, which has led to the development of mindfulness-based interventions (MBIs; Zhang, Lee, Mak, Ho, & Wong, 2021), which are therapeutic modalities that incorporate mindfulness as part of an integrated approach to treatment. These therapy modalities, often founded on mindfulness, emphasize the practice of mindfulness as a crucial component of both physical and mental well-being. Mindfulness-based stress reduction (MBSR), mindfulness-based cognitive therapy (MBCT), dialectical behavior therapy (DBT), and acceptance commitment therapy (ACT) are some examples of MBIs (Chiesa & Malinowski, 2011).

Mindfulness training supports a brain state conducive to emotional regulation and self-control (Chiesa, Calati, & Serretti, 2011; Friese, Messner, & Schaffner, 2012; Tang et al., 2010). Unlike CBT, which primarily aims to change or suppress specific thoughts or behaviors, MBIs emphasize cultivating a mental environment that allows for non-automatic responses to distressing internal experiences, such as cravings by teaching non-judgmental observation of thoughts, emotions, and bodily sensations (Shonin, Van Gordon, & Griffiths, 2014b; Wells, 2002). This aligns with the concept of state training, which aims to alter neural functioning to support executive control (Tang, Rothbart, & Posner, 2012, 2015). MBIs can reduce cravings in behavioral addictions by increasing awareness and cognitive control over automatic, urge-driven responses (Sancho et al., 2018). Practices such as ‘urge surfing’—a technique for observing and tolerating cravings without reactive engagement— may help individuals tolerate cravings without acting on them (Bowen et al., 2014; Witkiewitz, Bowen, Douglas, & Hsu, 2013). This mindful awareness disrupts habitual stimulus–response patterns and supports adaptive coping, thereby reducing relapse risk (Garland & Howard, 2018; Hsu & Forestell, 2021).

Such mechanisms are particularly relevant for GD where impulsivity, craving, and distorted cognitions are prominent. The existing body of research on mindfulness demonstrates that dispositional mindfulness (i.e., an individual's natural tendency to be aware and attentive to the present moment) is related to less severe problem gambling outcomes (de Lisle, Dowling, & Allen, 2012), and that developing mindfulness skills could help reduce impulsivity in stimulus-specific choices as assessed using delay discounting (Hendrickson & Rasmussen, 2013; Morrison & Heimberg, 2013; Yao et al., 2017).

To date, there is a scarcity of systematic reviews or meta-analyses conducted to determine the degree of effectiveness of mindfulness-based approaches for treating GD. The timeframe selected for the present systematic review, spanning from 2012 to 2025, was deliberately chosen to build upon and update the existing body of literature MBIs for GD, while also taking into consideration the relatively low number of publications that have examined this topic. A pivotal meta-analysis of 13 studies from 1989 to 2014 (Maynard, Wilson, Labuzienski, & Whiting, 2018) comprehensively examined studies up to that point, providing valuable insights into the efficacy of MBIs in treating GD. Since then, there have been significant advancements in both the conceptual understanding and practical application of MBIs within this domain. By focusing on studies conducted from 2012 onwards, the present review aimed to capture these recent developments, offering an updated synthesis of evidence that reflects the latest research trends, methodologies, and therapeutic outcomes in the field of mindfulness-based approaches to GD.

Therefore, the primary purpose of the present study was to gather the recent evidence in the extant literature regarding the efficacy of mindfulness as a fundamental or secondary factor in the treatment of individuals with varying degrees of problem gambling severity. Moreover, the review examined the degree to which MBIs are being implemented in a gambling setting in a methodical and quantitative manner, as well as to descriptively analyzing the different treatment and outcome components, in addition to gathering information regarding demographic samples previously underreported (individuals of Asian background and minors; Maynard et al., 2018) and examining the impact of these interventions on gambling-related outcomes. The specific research questions that the review attempted to answer were: (i) what is the current state of the literature—both in methodological quality and volume—investigating the effectiveness of MBIs in the treatment of gambling disorder?; and (ii) how do MBIs, relative to alternative treatments or no intervention, affect key domains of gambling-related symptomatology, including gambling frequency, severity, craving, urges, and maladaptive cognitive and emotional processes associated with gambling? In doing so, the review also considered the extent to which observed changes can be attributed to mindfulness-specific mechanisms as opposed to common therapeutic factors.

## Methods

The present systematic review was conducted and reported following the Preferred Reporting Items for Systematic Reviews and Meta-Analyses (PRISMA) guidelines (Moher et al., 2009) and was preregistered on the Open Science Framework (OSF) on June 12, 2024, to ensure transparency and reproducibility. The preregistration includes detailed information about the study objectives, methodology, and analysis plan, and is publicly accessible at https://doi.org/10.17605/OSF.IO/MVQZU.

### Search resources

The literature search was conducted by two independent reviewers and utilized five electronic databases: *PsycINFO, Scopus, EBSCO, PubMed,* and *Web of Science*. To build upon existing systematic reviews of similar scope, the search was focused on studies published from January 2012 to April 2025 wherever sources provided ‘filter by date’ functionality. As shown in Table 1, the search terms comprised a combination of terms and keywords that formed the Boolean operator that was used to search all five databases.

**Table 1.** Boolean search strategy for systematic review on mindfulness interventions for gambling disorders

Mindfulness	AND	intervention OR meditation OR treatment OR outcome OR program	AND	“gambling disorder” OR “gambling addiction” OR “pathological gambling” OR “problem gambling” OR “gamblers” OR “gambling dependence” OR “addictive gambling” OR “compulsive gambling” OR “disordered gambling” OR “excessive gambling” OR “impulsive gambling” OR “problematic gambling” OR “gambling”

*Note:* AND = a constraining; OR = an alternative-permitting Boolean operator.

### Eligibility criteria

The studies extracted from the datasets considered for the review had to meet several inclusion criteria. First, only studies conducted between January 2012 and April 2025 that examined the effects of MBIs among individuals with GD were included. Second, eligible studies had to utilize one of the following research designs: randomized controlled trials, quasi-experimental designs, single-group pre–post-test (SGPP) designs, or single-subject experimental designs. Third, MBIs in this context referred to approaches where participants were instructed or guided to focus their attention either on external stimuli (e.g., lights, sounds, or smells) or inwardly (e.g., thoughts, feelings, or urges), either as a primary or adjunctive therapy. Lastly, the studies had to employ mindfulness-based techniques aimed at fostering present-moment awareness of internal and external experiences, using both formal techniques (e.g., MBSR, ACT, MBCT, and DBT) and informal meditation exercises. Importantly, each study needed to evaluate the effects of these interventions on any measurable outcome. Studies were excluded if they were not published in English, were not published in peer-reviewed journals, or did not include a specific mindfulness-based intervention.

While the inclusion criteria permitted feasibility studies, case studies, and case series designs to provide a comprehensive overview of existing MBIs for gambling problems, studies with extremely small sample sizes and no inferential analysis were not included in the narrative synthesis or outcome-level comparison. These studies were retained for descriptive purposes to document emerging approaches and identify gaps in the literature, but their high risk of bias and lack of statistical power preclude meaningful contribution to the analysis.

### Study selection

Two authors screened all studies based on their titles and abstracts, followed by the full-text screening and data extraction which was checked by an additional author. In case of disagreement between reviewers, the relevant studies were discussed. No cultural or geographical limitations were placed in the selection process.

### Extracted information

The information extracted from each study included author names, year of study, country in which the study took place, sample size, the participant characteristics (gender, age, and diagnosis), method of intervention (including details about frequency, and duration of sessions for treatment versus control group), outcome measures and the instruments used to operationalize them, study design, and the findings (Table 2). Methodological inconsistencies, such as non-representative samples (Melero Ventola, Yela, Crego, & Cortés-Rodríguez, 2020; Shirk, Muquit, Deckro, Sweeney, & Kraus, 2022; van der Tempel et al., 2020) and differing control conditions, further limited comparability. Additionally, inconsistent statistical reporting made effect size aggregation unreliable. Given these challenges, a narrative synthesis was chosen to integrate findings while preserving the contextual nuances of each study.

**Table 2.** Characteristics of the included studies

Study	Intervention	Intervention description	Gambler-type comorbidity	*N*	Mean age (SD)	% Males	Study design	Main outcome measures	Major findings
1. Rolvien et al.(2024)	Self-guided internet-based intervention	12 modules addressing problematic and pathological gambling behaviors and associated emotional symptoms. Incorporated cognitive behavioral, mindfulness, and metacognitive strategies through text, video, and audio content. The modules are interactive, and users are advised to spend 30–60 min per module and to complete 2 modules weekly (module order can be freely chosen)	Problem gambling	**243**	*M* = 34.73, SD = 10.33	63.4%	RCT	PG-YBOCS, PHQ-9, SOGS, GABS	Significantly reduced gambling-related thoughts and behavior, depressive symptoms, and gambling severity in participants compared to a wait-listed control group. While the intervention showed moderate effect sizes for gambling symptom (PG-YBOCS) reduction (Cohen *d* = 0.59) and small effect sizes for depressive symptom (PHQ-9) reduction (Cohen *d* = 0.33), it did not yield significant improvements in gambling-specific dysfunctional thoughts
2. Turner et al. (2023)	Online group therapy	Eight weekly internet-based, therapist-guided 60–90-min sessions that included cognitive skills, mindfulness exercises, and mindful breathing. Each session starts with a mindfulness exercise. Each session began with a mindfulness exercise	Problem gambling	**10**	NA	50%	Pilot case study (SGPP)	PGSI, craving (a visual analogue scale), K6, REKT, MAAS	Clinically significant decreases in PGSI scores (*d* = −0.98), and at 12-month follow-up (*d* = −1.64). Cravings showed a small decrease from the pretest to post-treatment test of *d* = −0.30, and a large decrease of *d* = −0.80 at the 12-month follow-up. Both clients who completed treatment dropped below the threshold for severe gambling
3. Shirk et al. (2022)	MBRP	Nine sessions integrating mindfulness with cognitive behavioral approaches to increase awareness, acceptance, and self-compassion for cravings and urges	Gambling disorder	**3**	*M* = 50	100%	Case series, (SGPP)	Gambling frequency, cravings, CAMS-R, SUPPS-P	The post-treatment report of veterans showed less frequent engagement in their gambling behavior, fewer cravings, and less intense cravings. Increase in mindfulness, decrease in impulsivity measures
4. Bücker et al. (2021)	Self-guided internet-based intervention	Self-guided internet-based intervention, called Restart, adapted for pathological gambling problems over 8 weeks. Restart consists of 11 modules that involve gambling-related CBT techniques. The text-based program includes audio (e.g., mindfulness exercises) and psychoeducational videos (e.g., sleep disturbance models) to enhance engagement. Participants choose the module order, with a recommended pace of 1–2 per week (30–60 min each). Optional moderator support is available	Gambling Disorder	**150**	*M* = 35.03, SD = 11.27	67.30%	RCT	PG-YBOCS, PHQ-9, SOGS, GABS	No significant between-group differences were observed for all samples. Significant improvement was seen in the complete cases for both groups, with a strong effect size for the IG (*t*(30) = 5.05, *p* < .001, Cohen's *d* = −1.17) and a medium effect size for the CG (*t*(33) = 3.50, *p* = .001, Cohen's *d* = −0.72). Moderation analysis yielded significant improvements among older participants, those diagnosed with GD and no competing treatments compared to controls
5. van der Tempel et al. (2020)	Group-based MBI	Treatment was delivered in ten weekly 90-min group MBI sessions guided by a facilitator experienced in MBI. Focused on gambling triggers (e.g., seeing an advert for a casino, stress) and urges or actions that led to gambling	Gambling disorder	**9**	*M* = 56.2, SD = 1.84	0%	Repeated measures	NODS, PG-YBOCS, GCS, G-TLFB, GMQ, MAAS, SOGS, G-TLFB, GMQ, MAAS	Simple effects comparisons found a significant difference in mean PG-YBOCS scores for the first versus final treatment session, *F* (1, 8) = 9.36, *p* < .05 (effect size: partial *η*^2^ = 0.31). Significant reduction in craving from the beginning to the end of the *F* (1, 8) = 18.40, *p* < .01 (partial *η*^2^ = 0.37). 75% of the sample exhibited clinically significant reductions in PG-YBOCS and GCS
6. Shead et al. (2020)	Mindfulness-based meditation exercise	10-min audio guided the listener through basic mindfulness-based meditation techniques that included staying present, focusing on one's breath and attending to bodily sensations. Participants were sent a different audio stream each day for seven days plus an 8th and final one in the lab before post-assessment	Gambling craving	**59**	*M* = 21.6, SD = 4.4.	10%	RCT	Delay discounting task, GAQ, PGSI, GACS, MAAS, BIS	Gamblers in the meditation group demonstrated significant reductions in cravings pre- to post-mindfulness practice. There was no difference between delay discounting rates from part 1 to part 2 between the meditation practice condition and the audiobook control condition, as indicated by a non-significant interaction between time and condition. No significant difference in impulsivity
7. Melero Ventola et al. (2020)	MBCT	Mindfulness based-cognitive therapy intervention group (8 weeks) was compared to Mutual aid group intervention group (8 weeks) in the same sample, both interventions comprised of 8 weekly 2-h sessions	Gambling Disorder	**33**	*M* = 41.91, SD = 11.67	100%	Repeated measures	NORC Diagnostic Screen for GD, cravings, abstinence, FFMQ	The mutual-aid group intervention produced only moderate reductions in craving intensity (*η*^2^ = 0.27). In contrast, the MBCT program significantly increased the scores of the mindfulness-related variables (*η*^2^ ranging from 0.84 to 0.99) and reduced the craving intensity (*η*^2^ = 0.95), frequency (*η*^2^ = 0.93) and urge (*η*^2^ = 0.91)
8. McIntosh et al. (2016)	Manualized CBT, personalized CBT and MBI	Participants were randomized into three groups, the first two received manualized CBT and MBI in a cross-over design (MBT-first and CBT-first), the third group received personalized CBT. Treatment was delivered individually over an 8-week period, via face-to-face sessions, weekly. The initial assessment session lasted 90 min. Subsequent sessions lasted for 60 min	Problem gambling	**77**	*M* = 38.48, SD = 1.68	71.40%	RCT	DSM-V, SOGS, DASS-21, FFMQ, WBSI, RRQ	All three interventions tested returned large effect size improvements in PG behavior after seven sessions (Cohen's *d* range 1.46–2.01), at post-treatment and at 3 and 6-month follow-up. Mindfulness-first group showed significant improvements in gambling behaviors (Cohen's *d* = −1.69) as well as psychological distress (Cohen's *d* = −.76)
9. Dixon et al. (2016)	ACT	Treatment sessions were 60 min each, over eight weeks, and were developed from a gambling treatment model. During each treatment session, one or more of the six core ACT processes was incorporated to address self-generated rules related to the participant's gambling	Gambling disorder	**18**	*M* = 19.056 SD =.848	100%	RCT	Slot machine outcomes (wins, losses and near misses) and fMRI results, MAAS	The intervention group showed greater brain activation patterns for winning spins when compared to the initial scanning session, while participants in the control group showed no differentiation in brain activity following winning spins. Significant differences were found in participant responses to ACT treatment outcome measures the AAQ-II and MAAS, over time (AAQ-II approximate Cohen's *d* for ACT group was 0.98)
10. Chen et al. (2014)	MBCT, MBRP	learn and practice mindfulness awareness, to understand how mindfulness can be helpful to someone with problems related to gambling, and to enhance coping skills. 8-week program with two-hour sessions once a week	Community gamblers	**27**	*M* = 52.7, SD = 14.8	93%	Mixed methods	MAAS	The key result was that there was a statistically significant improvement in the levels of mindfulness of participants because of taking part in these groups (as indicated by the MAAS). The average change in scores was 0.75 (SD = .63) for Cohen's *d* of 1.18
11. Toneatto et al. (2014)	M-CBT	The experimental group received a five-session, manual-guided, mindfulness-enhanced group cognitive-behavioral therapy for problem gambling	Pathological gambling	**18**	*M* = 41.67 SD = 10.95	50%	Controlled pilot study	CAMH, DSM, GRCS, KIMS, BSI	The M-CBT group reported significantly fewer DSM gambling symptoms than the control group (for a Cohen's *d* of 1.319). Significant reductions between baseline and the three-month follow-up on gambling severity, psychiatric symptoms, and gambling urges (a Cohen's *d* of 1.32) were also found, indicating maintenance of the therapeutic gains
12. Christensen et al. (2013)	DBT	The group sessions followed nine sessions of a standard DBT format, and counsellors were instructed to follow a DBT orientation in their counselling sessions	Problem gambling	**14**	*M* = 46.5, SD = 11.6	31.80%	Proof of concept (SGPP)	PGSI, PAI-BOR, SBQ-R, K6, Gambling frequency and expenditure, CAMS-R	The intervention effectively reduced gambling behavior, with a third abstaining post-treatment and most reducing expenditure (83.3%) and sessions (53.9%). There are no statistically significant improvements in measures of gambling behavior. A small positive effect size was observed for gambling expenditure and psychological distress (with a Cohen's *d* of 0.30 and 0.38, respectively)

*Note:* BIS = Barratt Impulsiveness Scale, BPRS = Brief Psychiatric Rating Scale, BSI = Brief Symptom Inventory, CAMH = Centre for Addiction and Mental Health, CAMS-R = Cognitive and Affective Mindfulness Scale – Revised, CEQ = Craving Experience Questionnaire, CPGI = Canadian Problem Gambling Index, DASS-21 = Depression, Anxiety, and Stress Scale (21-item version), DSM = Diagnostic and Statistical Manual of Mental Disorders (version unspecified), DSM-IV = Diagnostic and Statistical Manual of Mental Disorders, Fourth Edition, DSM-IV-TR = Diagnostic and Statistical Manual of Mental Disorders, Fourth Edition, Text Revision, DSM-V = Diagnostic and Statistical Manual of Mental Disorders, Fifth Edition, FFMQ = Five Facet Mindfulness Questionnaire, GABS = Gambling Attitudes and Beliefs Survey, GACS = Gambling Abstinence Confidence Scale, GAF = Global Assessment of Functioning, GAQ = Gambling Abstinence Questionnaire, GCS = Gambling Craving Scale, GMQ = Gambling Motives Questionnaire, GRCS = Gambling-Related Cognitions Scale, G-SAS = Gambling Symptom Assessment Scale, G-TLFB = Gambling Timeline Followback, K6 = Kessler Psychological Distress Scale (6-item version), KIMS = Kentucky Inventory of Mindfulness Skills, MAAS = Mindful Attention Awareness Scale, MoCa = Montreal Cognitive Assessment, NODS = National Opinion Research Center DSM Screen for Gambling Disorders, NORC Diagnostic Screen for GD = National Opinion Research Center Diagnostic Screen for Gambling Disorders, PAI-BOR = Personality Assessment Inventory – Borderline Features Scale, PG-YBOCS = Pathological Gambling – Yale-Brown Obsessive Compulsive Scale, PGSI = Problem Gambling Severity Index, PHQ-9 = Patient Health Questionnaire (9-item version), REKT = (no standard full form identified), RRQ = Rumination-Reflection Questionnaire, SBQ-R = Suicidal Behaviors Questionnaire – Revised, SOGS = South Oaks Gambling Screen, SUPPS-P = Short UPPS-P Impulsive Behavior Scale, UPPS = Urgency, Premeditation, Perseverance, Sensation Seeking Impulsivity Scale, WBSI = White Bear Suppression Inventory.

### Quality assessment and risk of bias

The quality of the included studies was assessed using the Mixed Methods Appraisal Tool (MMAT) (Hong et al., 2018). This tool is particularly useful for evaluating the quality of different research methods in systematic reviews and meta-analyses. Two authors conducted the initial assessment, and any disagreements were resolved by consulting a third author. The assessment involved five specific questions tailored to each study's research design, with possible answers being ‘Yes’, ‘No’, or ‘Uncertain’ (Hong et al., 2018). Both authors worked together to evaluate each study, and their findings were discussed with the third author. Because the evaluations were done collaboratively, there was no interrater reliability index. Any differences were thoroughly discussed until a consensus was reached. It also included two initial questions to evaluate study clarity.

Afterwards, each study was assessed through five key guiding questions, with the MMAT offering distinct questions based on the research design. Studies assessed in the quantitative descriptive design category (Shirk et al., 2022; Shonin, Van Gordon, & Griffiths, 2014a; von Hammerstein et al., 2018) and the mixed methods category (Chen, Jindani, Perry, & Turner, 2014) consistently received ‘Yes’ responses for all quality criteria questions. Similarly, most studies evaluated under the quantitative nonrandomized criteria received ‘Yes’ responses, except for three studies where assessors marked uncertainty regarding the accounting for confounders in the design and analysis (Christensen et al., 2013; Toneatto, Pillai, & Courtice, 2014; Turner et al., 2023), and one study with incomplete outcome data (van der Tempel et al., 2020). For the five quantitative randomized controlled trials (RCTs) included in the sample, most quality criteria questions were answered ‘Yes’. However, some studies lacked a detailed description of the randomization process (Dixon, Wilson, & Habib, 2016; McIntosh, Crino, & O'Neill, 2016; Shead, Champod, & MacDonald, 2020), and one did not provide information on blinding (McIntosh et al., 2016).

## Results

### Study selection and design

**Fig. 1.**
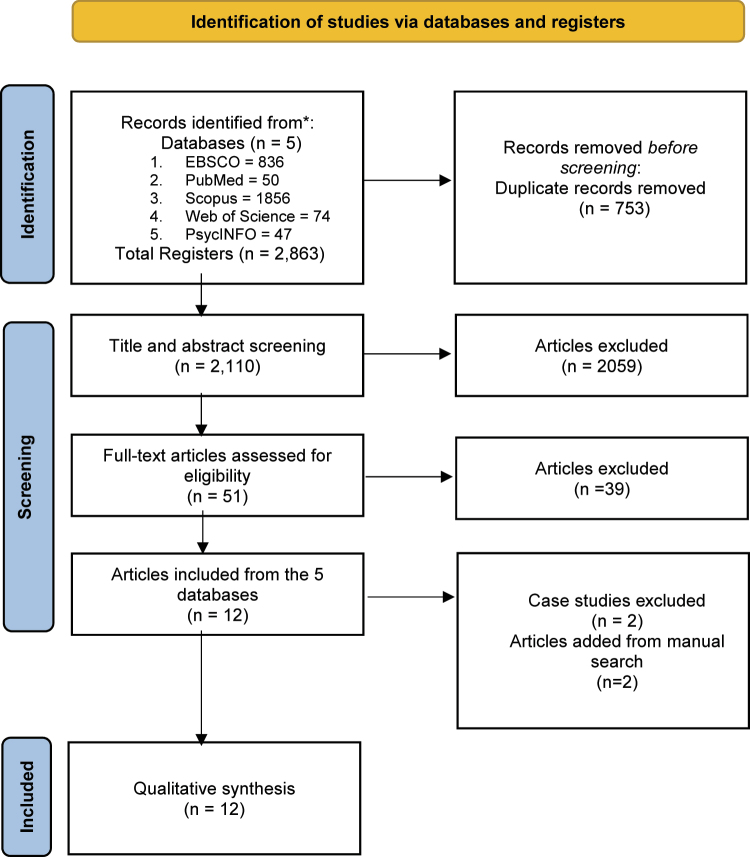
Flow chart of the selection process. From Page et al. (2021)

Initially, the search identified 2,863 outputs (see the study selection flow chart in Fig. 1). Upon removing duplicated records, 2,110 full-text outputs went through a title and abstract screening for eligibility, and 2,059 of them were excluded for several reasons (e.g., because they were not peer-reviewed [e.g., books, review papers], the study did not include a mindfulness intervention, the sample did not include individuals who gambled). Subsequently, 51 papers were assessed more thoroughly. After examining the full text of these, 39 were excluded because of a lack of focus on mindfulness in the intervention, irrelevant population, unclear or insufficient methodological details, and inappropriate study design.

A total of 12 studies initially met the eligibility criteria and were included for full review. However, two of these (Shonin et al., 2014a; von Hammerstein et al., 2018) were later excluded from the analysis due to being single-case studies with limited generalizability and increased risk of bias. Two additional studies (i.e., Rolvien et al., 2024; Turner et al., 2023) were identified through a targeted *Google Scholar* search and included in the synthesis.

The final sample of 12 included studies comprised five randomized controlled trials (RCTs): Bücker, Gehlenborg, Moritz, and Westermann (2021; *N* = 150), Dixon et al. (2016; *N* = 18), McIntosh et al. (2016; *N* = 77), Shead et al. (2020; *N* = 59), and Rolvien et al. (2024; *N* = 243); two repeated-measures designs: Melero Ventola et al. (2020; *N* = 33) and van der Tempel et al. (2020; *N* = 9); one controlled but nonrandomized pilot study: Toneatto et al. (2014; *N* = 18); one mixed-methods study: Chen et al. (2014; *N* = 27); and three single-group pretest–posttest studies: Turner et al. (2023; *N* = 10), Shirk et al. (2022; *N* = 3), and Christensen et al. (2013; *N* = 14). Of these, 10 were included in the narrative synthesis and analysis while one pilot study and one feasibility study (Shirk et al., 2022; Turner et al., 2023) were retained for explorative purposes only due to the absence of inferential statistics and extremely limited sample sizes.

### Mindfulness components across interventions

The mindfulness components implemented across the included studies varied in format, intensity, and theoretical emphasis, although specific practices were recurrent. Present-moment awareness and breath-focused attention featured prominently in several interventions (Toneatto et al., 2014; Shead et al., 2020). Urge surfing was reported by Melero Ventola et al. (2020) and van der Tempel et al. (2020), where it corresponded with large reductions in craving intensity and frequency. McIntosh et al. (2016) incorporated a broad suite of MBCT-based elements, including psychoeducation, mindful eating, brief breathing practices, strategies for managing emotional discomfort during meditation, and cultivating awareness of automatic thoughts.

Chen et al. (2014) integrated the SOBER technique (Stop, Observe, Breathe, Expand, Respond) into a MBCT and MBRP-informed protocol. Christensen et al. (2013) employed DBT to teach present-focused awareness and distress tolerance. Dixon et al. (2016) delivered mindfulness as part of an ACT-based intervention focused on helping participants observe thoughts, bodily sensations, and emotions in real time to distinguish mindful from automatic behavior. Bücker et al. (2021) and Rolvien et al. (2024) included mindfulness-based relaxation exercises such as breath awareness, body scans, and yoga elements in an internet-based intervention program called ‘Restart’. Overall, more comprehensive interventions that combined multiple components—including meditation, psychoeducation, and experiential exercises—were associated with more robust and sustained improvements in mindfulness scores and gambling-related outcomes (e.g., McIntosh et al., 2016; Melero Ventola et al., 2020).

### Outcome measures

The studies varied in the target outcome and measures to assess these outcomes. Various problem gambling behavior assessment instruments were used in the included studies. The most commonly used instruments in the sample were the Diagnostic and Statistical Manual of Mental Disorders (DSM-IV and DSM-5) (*n* = 4), Problem Gambling Severity Index (PGSI; Ferris & Wynne, 2001) (*n* = 3), South Oaks Gambling Screen (SOGS; Lesieur & Blume, 1987) (*n* = 4), and pathological gambling adaptation of the Yale-Brown Obsessive-Compulsive Scale (PG-YBOCS; Pallanti, DeCaria, Grant, Urpe, & Hollander, 2005) (*n* = 3). Regarding the instruments used to assess mindfulness (in the context of the present review, the focus was on *trait* mindfulness; Carpenter, Conroy, Gomez, Curren, & Hofmann, 2019), the most commonly used measurements were the Mindful Attention Awareness Scale (MAAS; Brown & Ryan, 2003) (*n* = 6), and the Five Facet Mindfulness Questionnaire (FFMQ; Baer et al., 2008) (*n* = 3).

All studies included in the systematic review used outcome measures related to gambling behavior except for one (Chen et al., 2014). It focused on the improvement in trait mindfulness and utilized qualitative and quantitative measures to examine the effects of mindfulness-based cognitive therapy (MBCT) and mindfulness-based stress reduction (MBRP) on the levels of mindfulness among participants as a result of participating in the group interventions. In this study, Chen et al. reported a statistically significant improvement in the Mindful Attention Awareness Scale (MAAS) scores of the participants from pre- to post-treatment. The change in scores yielded a large effect size (Cohen's *d* of 1.18). Results from a course reflection questionnaire corroborated these findings with seven themes emerging from the feedback collected, six of which cited improvements in participants' lives.

### Randomized controlled trials

McIntosh et al. (2016) investigated how combining a mindfulness-based treatment with manualized CBT for individuals with gambling problems influenced treatment outcomes when compared to case formulation-driven CBT (personalized), and whether the order in which the interventions were delivered influenced their effectiveness. The mindfulness components, adapted from MBCT, included psychoeducation on mindfulness for problem gambling, mindful eating, increasing awareness of automatic thoughts, insight meditation, brief breathing practices, managing difficult emotions during meditation, and strategies to overcome barriers to regular practice. All three groups (first group had MBI followed by manualized CBT, second group had CBT first followed by MBI, and the control group had personalized CBT) demonstrated large reductions in DSM-5 gambling symptoms post-treatment, sustained at three- and six-month follow-ups.

Key findings suggested that both the mindfulness-based and the ‘treatment as usual’ (TAU) interventions effectively reduced PG behavior and distress, with additional benefits in measures of quality of life and mindfulness, surpassing the manualized CBT utilized in this study. Regarding the effects of sequencing, the MBI-first group showed the largest effect size improvements in PG behavior and several secondary measures at post-treatment (e.g., mindfulness facets such as “Act with awareness” had a Cohen's *d* of 1.06). The CBT-first group also displayed significant improvements in PG behavior, but the addition of mindfulness training after CBT produced enhanced results in secondary outcomes such as mindfulness, thought suppression, and quality of life (QOL) mental functioning. However, quality of life improvements were evident only after the mindfulness phase in the CBT-first group.

Shead et al. (2020) tested the efficacy of a brief mindfulness meditation intervention, incorporating foundational mindfulness meditation techniques involving present-moment awareness, focused attention on the breath, and heightened awareness of bodily sensations, in curbing gambling cravings. The study utilized a randomized design with an active control group. Individuals who exhibited high levels of inherent mindfulness traits (i.e., scored high on a dispositional mindfulness scale) tended to be less impulsive as reported using the Barratt Impulsiveness Scale (BIS; Patton, Stanford, & Barratt, 1995), although the 10-min daily audio-guided mindfulness meditation exercises administered for a week (while the control group listened to an audiobook) in their RCT showed no significant effects on impulsivity. A significant interaction between time and condition was found, which showed that gamblers who engaged in daily mindfulness meditation for a week experienced fewer gambling cravings. In contrast, the active control group, who did not practice meditation, experienced no change in their cravings. The meditation intervention did not significantly affect delay discounting in comparison to the control group, nor did the delay discounting scores relate to gambling involvement.

Neurological evidence for the benefits of ACT (of which mindfulness is a core factor) among a group of disordered slot machine gamblers was reported in a study conducted by Dixon et al. (2016). In their study, they set out to understand the brain-behavior relationships that emerged following behavioral therapy (in this case, the treatment was eight weeks of 60-min ACT sessions). Participants were randomized to either the ACT group or a no-treatment control group, and were assessed while they gambled using an fMRI scanner before and after treatment. The mindfulness component of the ACT interventions involved helping participants distinguish mindful from mindless behavior by observing their thoughts, emotions, and bodily sensations in real time. The study showed that following the ACT intervention, participants exhibited increased blood-oxygen-level dependent (BOLD) activity in the anterior cingulate cortex (ACC) and ventromedial prefrontal cortex (vmPFC) in response to winning outcomes compared to baseline measurements. In contrast, the control group's brain activity for winning spins did not change. The change found among those in the treatment group mirrored the brain activation seen among individuals without gambling problems. Over time, the treatment group reported significantly different ratings for winning and losing outcomes, whereas those in the control group did not show any such differences. Significant differences were also found in mindfulness as assessed using MAAS scores where following treatment, participants reported more mindfulness-related behaviors than the control group.

Bücker et al. (2021) evaluated the potential of a self-guided internet-based intervention that included mindfulness-based and metacognitive techniques. Participants were randomized to either the intervention or a wait-list control group with access to treatment as usual. The study's main aim was to evaluate the efficacy of an internet-based intervention program called ‘Restart’ (implemented over an eight-week period) in reducing symptoms of GD. The mindfulness module focused on the presentation of mindfulness-based relaxation and attention exercises, such as relaxation and breathing exercises, body scan, and yoga elements. No significant between-group effects were found for the primary outcome (gambling severity; PG-YBOCS) in any analytic sample (*p* > .05). However, within-group improvements were significant in both arms, with a large effect in the intervention group (*t*(30) = 5.05, *p* < .001, *d* = −1.17) and a medium effect in the control group (*t*(33) = 3.50, *p* = .001, *d* = −0.72). Depression, which was measured using the Patient Health Questionnaire-9 (PHQ-9; Kroenke, Spitzer, & Williams, 2001), declined significantly among those in the intervention group (*d* = −0.43), and both groups showed large improvements in gambling severity assessed by the SOGS (IG: *d* = −1.11; CG: *d* = −1.17). Gambling-related cognitions (GABS; Breen & Zuckerman, 1999) improved among those in the control group (*d* = −0.42), with a trend toward significance among those in the intervention group (*p* = .057, *d* = −0.32). Moderation analyses showed greater benefit among participants with higher baseline gambling and depression scores, comorbid anxiety, or higher program satisfaction.

Rolvien et al. (2024; *N* = 243) conducted a randomized controlled trial (RCT) investigating the efficacy of the internet-based ‘Restart’ program. Compared to a wait-list control group, participants in the intervention group exhibited significantly greater reductions in gambling-related thoughts and behaviors (PG-YBOCS; *d* = 0.59), gambling severity (*d* = 0.40), and depressive symptoms (PHQ-9; *d* = 0.33). No significant improvement was observed in gambling-related cognitive distortions (GABS; *d* = 0.23, *p* = .07). Treatment effects were stronger among participants with higher baseline severity and positive treatment expectations.

### Non-randomized controlled trial

Toneatto et al. (2014) investigated the efficacy of a five-session group mindfulness intervention integrated into CBT (mindfulness-enhanced cognitive behavioral treatment; M-CBT) among 18 individuals diagnosed with GD. The intervention focused on using the breath to anchor present-moment awareness, noticing gambling-related thoughts, and redirecting attention non-reactively. While this study had a non-randomized wait-list control group and therefore does not meet the criteria to be classified as an RCT, it reported significant reductions in reported DSM gambling symptoms among those in the M-CBT group at post-treatment assessment compared to the active control group. Additionally, at the three-month follow-up, those in the M-CBT group scored significantly lower on gambling severity, gambling urges, and psychiatric symptoms. Moreover, at the three-month follow-up, the proportion of participants who met diagnostic criteria for GD significantly dropped to 21% compared to baseline (95%). Importantly, follow-up outcomes were influenced by continued mindfulness practice. Among the 14 participants assessed at follow-up, those who reported mindfulness practice (*n* = 9) demonstrated markedly better outcomes than non-practitioners (*n* = 5): significantly lower gambling urges (*t*[12] = 3.34, *p* < .01), reduced psychiatric symptoms (*t*[12] = 2.81, *p* < .02), and a trend toward fewer DSM gambling symptoms (*t*[12] = 1.98, *p* < .07).

### Repeated measures studies

A study by Melero Ventola et al. (2020) investigated the effectiveness of a mutual-aid group intervention and an MBCT training group in a repeated-measures within-participants design with six assessment time points: baseline (T1), post-mutual-aid group before MBCT (T2), post-MBCT (T3), one-month follow-up (T4), three-month follow-up (T5), and six-month follow-up (T6). The intervention included mindfulness exercises and imaginal exposure through a “surfing the craving” practice, aimed at increasing tolerance of urges. Following the mutual-aid intervention (T1–T2), small to moderate improvements were observed in craving intensity (*η*^2^ = .27) and mindfulness total scores (*η*^2^ = .43), with negligible or non-significant changes in other craving dimensions. In contrast, large improvements were observed from pre- to post-MBCT (T2–T3) in craving intensity (*η*^2^ = .96), frequency (*η*^2^ = .93), urgency (*η*^2^ = .91), and total craving (*η*^2^ = .96), alongside substantial increases in mindfulness total scores (*η*^2^ = .99) and all five mindfulness facets (*η*^2^ range = .84–.99). These gains remained stable at one-, three-, and six-month follow-ups, although slight declines were observed in the “observing” and “acting with awareness” subscales. No participant relapsed during MBCT or follow-up. All participants reported engaging in mindfulness practice post-intervention, with high satisfaction (*M* = 9.33, SD = 0.64) on a 1–10 scale, where higher scores indicate greater satisfaction.

van der Tempel et al. (2020) also reported significant results that provided preliminary support for the efficacy of stand-alone MBI on treatment-resistant gamblers, who served as their own controls. The MBI emphasized present-moment awareness and nonjudgmental self-examination, with a GD-adapted version specifically targeting gambling triggers and urges. Scores on the PG-YBOCS during alternating MBI sessions showed a significant decrease over sessions, with a notable difference observed between the first and final treatment sessions. Most cases showed a clinically significant decrease, with a 26% decrease in PG-YBOCS scores ($\eta^{2}$ = 0.31). In addition, six out of the eight participants who completed the study exhibited clinically significant reductions in PG-YBOCS scores and Gambling Craving Scale (GCS) scores.

### Single group pretest posttest (SGPP)

Christensen et al. (2013) evaluated a modified DBT treatment program for individuals with gambling problems in a group format. However, the design lacked a control group. The program lasted for nine weeks, and its main objective was to assist individuals who gambled to gain applicable mindfulness skills (e.g., being aware of the present moment and judging less) that would aid in reducing problem gambling severity (as assessed using the PGSI). By the end of the program, 10 of the 14 participants (83.3%) who completed the program, and the pre- and post-program survey were either abstinent or had reduced their gambling expenditure. However, these results were not statistically significant. Psychological distress significantly decreased pre- to post-treatment at the group level (*p* = .05), as well as significant improvement in mindfulness and distress tolerance.

Two studies employed small-sample, single-group pre–post designs and were excluded from the main synthesis due to high risk of bias and insufficient sample size. Shirk et al. (2022) conducted a feasibility case series of mindfulness-based relapse prevention (MBRP) with three U.S. veterans, reporting reductions in monthly gambling behavior and cravings, as well as increased mindfulness and improved emotion regulation and impulsivity. However, outcomes were not statistically analyzed.

Turner et al. (2023) evaluated a therapist-guided internet-based intervention integrating mindfulness and cognitive skills. Of 24 eligible participants, only two completed the program and a total of ten completed the follow-up assessment. PGSI scores decreased from 15 (severe gambling) to 9 at post-treatment (*d* = −0.98), and to 5 at 12-month follow-up (*d* = −1.64), with concurrent reductions in cravings (*d* = −0.30 to −0.80). Although changes were clinically notable, the sample size precluded inferential analysis.

### Effect sizes

The effect sizes reported in the included studies varied widely, reflecting the differences in intervention types, combinations, durations, and populations, as well as the heterogeneity in study design and methodology. Most studies reported medium to large effect sizes, particularly in interventions that combined mindfulness with cognitive-behavioral strategies. A weighted average effect size of Cohen's *d* = 0.76 was calculated across five studies assessing gambling severity using measures such as the SOGS, PGSI, PG-YBOCS, DSM-based criteria, and GABS. The included studies were those by Rolvien et al. (2024, *N* = 243), Bücker et al. (2021, *N* = 150), McIntosh et al. (2016, *N* = 77), Toneatto et al. (2014, *N* = 18), and Christensen et al. (2013, *N* = 14), yielding a total sample size of 502. This estimate represents a pooled indicator of gambling severity reduction associated with MBIs across diverse study contexts. However, given variability in study designs, outcome measures, and populations, it should be interpreted as a general index of treatment impact rather than a precise quantification of efficacy. The largest effect sizes were demonstrated in two RCTs (i.e., McIntosh et al., 2016; Toneatto et al., 2014) both of which used mindfulness integrated into a CBT protocol.

## Discussion

The present systematic review evaluated the efficacy of MBIs in addressing the dysfunctional attitudes, thoughts, and behaviors characteristic of gambling disorder (GD) and problem gambling. The analysis of 12 studies, encompassing a variety of designs including RCTs, repeated measures studies, mixed-methods, and single-group pretest posttest studies found compelling evidence that MBIs hold significant potential as a therapeutic approach. Across all the studies that assessed gambling frequency (*n* = 6), MBIs were shown to effectively decrease it. These results were maintained at three-month (McIntosh et al., 2016; Melero Ventola et al., 2020; Toneatto et al., 2014), six-month (McIntosh et al., 2016; Melero Ventola et al., 2020), and 12-month (Turner et al., 2023) follow-ups, in all the studies where follow-up assessments were performed, suggesting that MBIs may offer a durable strategy for managing GD symptoms. However, the heterogeneity of study designs, variability in mindfulness components, small sample sizes, and methodological limitations necessitate a nuanced interpretation of these results. Therefore, the following discussion synthesizes the primary takeaways, situates them within the existing literature on addiction and mindfulness, and draws systematic inferences about the applicability of MBIs for problem gambling, while integrating original conclusions derived from a detailed examination of the 12 studies.

Six out of the ten studies included in the narrative synthesis reported reductions in gambling cravings (Bücker et al., 2021; Christensen et al., 2013; McIntosh et al., 2016; Melero Ventola et al., 2020; Shead et al., 2020; van der Tempel et al., 2020). Shead et al. (2020) found that a brief one-week mindfulness meditation reduced cravings, although its failure to impact impulsivity and delay discounting—namely, the mental simulation and valuation of delayed rewards highlighted a potential limitation in generic interventions that do not target specific mechanisms of gambling. Additionally, mindfulness meditation cultivates nonjudgmental, present-moment awareness, which may inadvertently sideline the future-oriented cognition needed to reduce delay discounting. In contrast, episodic future thinking, which explicitly prompts individuals to vividly imagine personal future events, robustly lowers discount rates by engaging hippocampal and prefrontal networks involved in planning and valuation (Peters & Büchel, 2010). Brief mindfulness interventions have yielded only modest, short-lived reductions in delay discounting (Ge et al., 2025; Shead et al., 2020), suggesting that without actively invoking future simulations, mindfulness alone may be insufficient to decrease impulsive preference for immediate rewards.

Secondary outcomes, such as reductions in psychological distress and increases in trait mindfulness, were frequently observed (e.g., Chen et al., 2014, *N* = 27; Melero Ventola et al., 2020, *N* = 33). Psychological distress outcomes were examined in multiple studies. Bücker et al. (2021; *N* = 150) observed significant reductions in depression and anxiety symptoms, while Christensen et al. (2013; *N* = 27) and Rolvien et al. (2024; *N* = 243) reported concurrent declines in general emotional distress. These results suggest that MBIs may target transdiagnostic mechanisms such as avoidance, suppression, and hypervigilance—processes also identified by Dudley, Kuyken, and Padesky (2011) and Petry (2005) as maintaining factors in comorbid GD and mood disorders. This is particularly relevant given the high comorbidity rates reported across study samples.

A notable trend across the reviewed RCTs (Bücker et al., 2021, *N* = 150; Dixon et al., 2016, *N* = 18; McIntosh et al., 2016, *N* = 77; Rolvien et al., 2024, *N* = 243; Shead et al., 2020, *N* = 59) was the consistent reduction in GD behavior and symptomatology, alongside improvements in mindfulness and emotional regulation. The addition of mindfulness teaches individuals with gambling problems to modify their responses to gambling-related thoughts rather than to try to dismantle or change strongly embedded and rigid beliefs and thought patterns, which is consistent with findings in previous studies (de Lisle et al., 2012; Toneatto et al., 2007). Melero Ventola et al. (2020) also found that MBCT was more effective after a mutual aid group at decreasing gambling cravings than a mutual-aid group intervention by itself. This shift in approach from ‘controlling the urge’ to ‘surf the craving’ highlights the adjunctive benefit of MBIs.

The potential additive value of mindfulness-based components appears most evident when mindfulness supports, rather than replaces, cognitive-behavioral aspects of treatment. In the study by McIntosh et al. (2016), all three treatment conditions produced large and sustained reductions in gambling severity. However, secondary outcomes such as rumination and mental-health-related quality of life and rumination improved primarily after participants completed the mindfulness phase, suggesting that mindfulness may enhance CBT's effects on transdiagnostic processes. Notably, groups received the same number of sessions, indicating that any differences in outcome were not due to greater treatment exposure. Toneatto et al. placed the mindfulness after the cognitive-behavioral part of the intervention, which showed significant reductions in DSM gambling severity symptoms and urges, with gains maintained at three-month follow-up (Toneatto et al., 2014). What further highlighted the additive value of mindfulness components is that participants who continued mindfulness practice after treatment achieved significantly better clinical outcomes at follow-up than those who did not. However, these results cannot be conclusively ascribed to the mindfulness component, given that it was delivered as an adjunct to CBT, an already well-established and empirically supported treatment.

These findings are consistent with and extend the broader literature on the use of MBIs in addiction treatment. The therapeutic shift from ‘controlling the urge’ to ‘surfing the craving’ (Marlatt, Bowen, Chawla, & Witkiewitz, 2004) could explain the adjunctive benefit of MBIs by enhancing awareness of triggers (Bowen et al., 2014). Melero Ventola et al. (2020) similarly found that MBCT was more effective at decreasing gambling cravings when offered after a mutual-aid group than the mutual-aid group alone. The potential of MBIs to augment existing treatments is further supported by findings from Christensen et al. (2013), who reported that a combination of MBI and DBT among a cohort of problem gamblers (*N* = 14, 78.6% female) resulted in abstinence or a reduction in problem expenditure for 83% of completers, while simultaneously increasing distress tolerance.

Several studies explored the efficacy of structure, multi-component internet-delivered MBIs (Bücker et al., 2021; Rolvien et al., 2024; Turner et al., 2023). Bücker et al. (2021) found that a self-guided internet-based program was feasible and well-accepted, showing significant improvements in gambling symptoms for some subgroups, although it did not outperform the control group in the intent-to-treat analysis. Rolvien et al. (2024) reported that a self-guided internet-based intervention, which included mindfulness elements, led to greater reductions in gambling symptoms and depressive symptoms compared to wait-list controls, especially for those with higher symptom severity and positive treatment expectations. These suggest that personalized support may be an important factor of online MBI success, a conclusion which is supported by Andersson et al.'s (2014) findings on the efficacy of therapist-supported digital mental health tools. Overall, while these online mindfulness-augmented approaches appear to be feasible and may suggests a scalable model for reaching the low-treatment-seeking population, which constitutes a significant barrier to care (Gainsbury, Hing, & Suhonen, 2014; Håkansson & Widinghoff, 2020), more robust, large-scale research is needed to confirm their general efficacy for treating GD.

The neurological shift noted by Dixon et al. (2016) resonates with Garland et al.'s (2014) work on mindfulness attenuating reward sensitivity in addiction, suggesting that MBIs may disrupt the reinforcement cycles central to GD. In their ACT-based protocol, participants exhibited increased activation in the ACC and vmPFC during exposure to gambling outcomes. These patterns resembled non-disordered controls and are implicated in cognitive control and emotion regulation, supporting the notion that mindfulness enhances regulation of reward-related reactivity. This aligns with Garland et al.'s (2014) model suggesting that mindfulness reduces affective bias toward salient cues by modulating default reward processing networks, thereby disrupting addictive feedback loops.

Overall, while CBT remains the empirically strongest intervention for immediate therapeutic effect, MBIs show comparable effect sizes and demonstrate specialized strength in long-term outcomes and emotional regulation. According to a recent meta-analysis by Pfund et al. (2023), CBT exhibits a large immediate effect on reducing gambling disorder severity (*g* = − 0.91) at post-treatment relative to control. MBIs in the present sample, by comparison, achieved a robust moderate-to-large, pooled effect size of Cohen's *d* = 0.76, where effect sizes were coded so that positive values indicated improvement. A key differentiation arises at follow-up: studies consistently report that CBT interventions show lack of significant long-term effects on gambling outcomes (Pfund et al., 2023), while MBI studies indicate the maintenance or strengthening of therapeutic gains up to 12 months (Turner et al., 2023). Moreover, MBIs demonstrate a specific advantage in treating transdiagnostic factors such as craving (Shead et al., 2020; Turner et al., 2023; van der Tempel et al., 2020), yielding pooled moderate-to-large effects on gambling cravings, in addition to rumination, thought-suppression, and distress tolerance. Conversely, motivational interviewing as a standalone treatment shows limited and uncertain overall effectiveness (Cowlishaw et al., 2012). Therefore, integration of mindfulness with CBT harnesses the strong behavioral change effects of CBT and the emotional regulation strengths of MBIs, offering a more comprehensive approach for long-term recovery.

Despite promising results, it is essential to contextualize these findings within the broader psychotherapy literature. A substantial body of evidence, including work by Wampold (2015) and Cuijpers, Reijnders, and Huibers (2019), has shown that common factors—such as the therapeutic alliance, treatment credibility, and client expectancy—account for a large proportion of treatment outcomes, often rivaling or exceeding the contribution of intervention-specific techniques. These common factors should therefore be considered when interpreting the improvements reported across MBI studies because some of the positive effects may not be attributable solely to mindfulness mechanisms. To validate mindfulness as a true mechanism of change which drives therapeutic outcome, it must meet several criteria such as temporal precedence, dose-response, and accounting for confounding variables.

The findings reported by Toneatto et al. (2014) provide preliminary evidence that continued mindfulness practice may contribute to improved clinical outcomes in problem gambling because participants who engaged in mindfulness post-treatment reported significantly lower gambling urges and psychiatric symptoms, and a trend toward fewer DSM gambling symptoms. These findings point to a possible dose-response relationship and temporal precedence, two of the five requirements listed by Cuijpers et al. (2019) as being essential to proving a causal treatment mechanism.

However, the study's design limitations temper this interpretation. The small sample size (*N* = 18), use of a waitlist control rather than an active comparator, lack of randomization at follow-up, reliance on self-reported mindfulness practice, and absence of direct behavioral measures of gambling all constrain the strength of causal claims. A particular challenge lies in the variability of mindfulness components across studies. Some interventions relied on brief guided practices (Shead et al., 2020), whereas others used multi-component MBCT protocols with psychoeducation, mindful eating, and emotional regulation strategies (McIntosh et al., 2016). Additional elements included urge surfing (Melero Ventola et al., 2020; van der Tempel et al., 2020), body-focused awareness in ACT (Dixon et al., 2016), and distress-tolerance practices within DBT (Christensen et al., 2013). Hybrid formats such as mindfulness-enhanced CBT (Toneatto et al., 2014) and online programs (Bücker et al., 2021; Rolvien et al., 2024) added further diversity, often with limited detail on delivery. This diversity complicates interpretation and makes it difficult to determine whether observed improvements are attributable to mindfulness-specific mechanisms, particular components, or broader therapeutic factors.

As Cuijpers et al. (2019) emphasize, most psychotherapy research—including the studies in the present review—is correlational in nature, meaning that observed improvements cannot be definitively attributed to mindfulness practice alone. Other common therapeutic factors, such as expectancy effects or therapist support, may have contributed to the outcomes. Therefore, while the studies in the present review offer promising data that support mindfulness as a potential specific mechanism of change, these findings should be interpreted as exploratory and warrant replication through rigorous, controlled trials designed to isolate and test mindfulness-specific effects. Moreover, few studies assessed participants' baseline mindfulness levels, treatment adherence, or engagement with specific mindfulness components—factors essential to evaluating treatment dosage and the mechanism of action. Consequently, it remains unclear to what extent observed improvements are attributable to mindfulness-specific processes versus broader therapeutic or expectancy effects.

Several other limitations constrain the interpretability and generalizability of the present review. First, the included studies exhibited substantial methodological heterogeneity in both design and outcome measures. This precluded a meta-analytic synthesis and necessitated a narrative approach. Most samples were small, with few studies adequately powered to detect reliable effects. Although case studies were excluded from the main synthesis, their initial inclusion underscores the scarcity of large-scale trials and the early stage of this literature.

Second, statistical reporting was often incomplete or inconsistent. Key metrics such as standard deviations, effect sizes, and follow-up data were frequently missing or heterogeneously reported, hindering cross-study comparability and robust estimation of intervention effects. Additionally, many studies failed to describe critical methodological safeguards, such as randomization procedures, allocation concealment, blinding, or control of confounding variables, increasing the risk of bias.

Third, inconsistencies in reporting and methodological heterogeneity complicate interpretation. Not all studies clearly specified the type of control group used (e.g., waitlist, treatment-as-usual [TAU], or active control), making it difficult to assess the internal validity of their findings. The exploratory studies (Shirk et al., 2022; Turner et al., 2023) employed single-group or underpowered designs, precluding statistical testing and limiting generalizability despite clinically promising findings.

Fourth, the demographic and clinical profiles of the study participants were predominantly adult males with diagnosed GD. While this could indicate that MBIs are effective for this subgroup, particularly those with moderate to severe symptoms, it also limits generalizability to females, adolescents, and diverse ethnic groups, and presents a risk of bias. This is a critical need given GD's chronicity (Williams & Williams, 2025).

While the present review provides valuable insights into MBIs for gambling disorders, the process itself carries limitations. The search was confined to five major databases, potentially missing studies in less mainstream outlets or non-English publications. Additionally, the reliance on published literature may have overrepresented positive outcomes because negative or null findings are less likely to reach publication. The narrative synthesis, while appropriate given study heterogeneity, lacked the quantitative rigor of a meta-analysis, introducing a degree of interpretive subjectivity.

### Clinical implications

Clinically, MBIs offer a promising adjunct to existing treatments, particularly for those hesitant to seek help. Their ability to reduce cravings and distress (Toneatto & Gunaratne, 2009) could be leveraged through group or online platforms, with Turner et al.'s (2023) success suggesting that therapist guidance enhances outcomes. Policymakers might consider subsidizing guided online MBIs or integrating mindfulness training into therapist curricula, especially for rural or underserved areas.

## Conclusion

The present review identified preliminary but promising trends in MBIs reducing gambling frequency, cravings, and psychological distress among individuals with GD, especially when combined with CBT or delivered through guided digital formats. However, consistent with broader psychotherapy research, these benefits might not be solely due to mindfulness mechanisms. Future research should explicitly consider general therapeutic factors, employ adequately powered RCTs with active controls, include larger and more diverse samples—such as females and different socioeconomic groups (Welte et al., 2006)—and use longitudinal designs to evaluate long-term effectiveness. Until then, MBIs ought to be viewed as a potentially valuable element within a broader, integrative treatment framework for gambling disorder.
